# Supplementary material for: Famotidine activates the vagus nerve inflammatory reflex to attenuate cytokine storm
Source: Mol Med. 2022 May 16;28:57. doi: 10.1186/s10020-022-00483-8 (PMC9109205; doi:10.1186/s10020-022-00483-8)

Figure 1

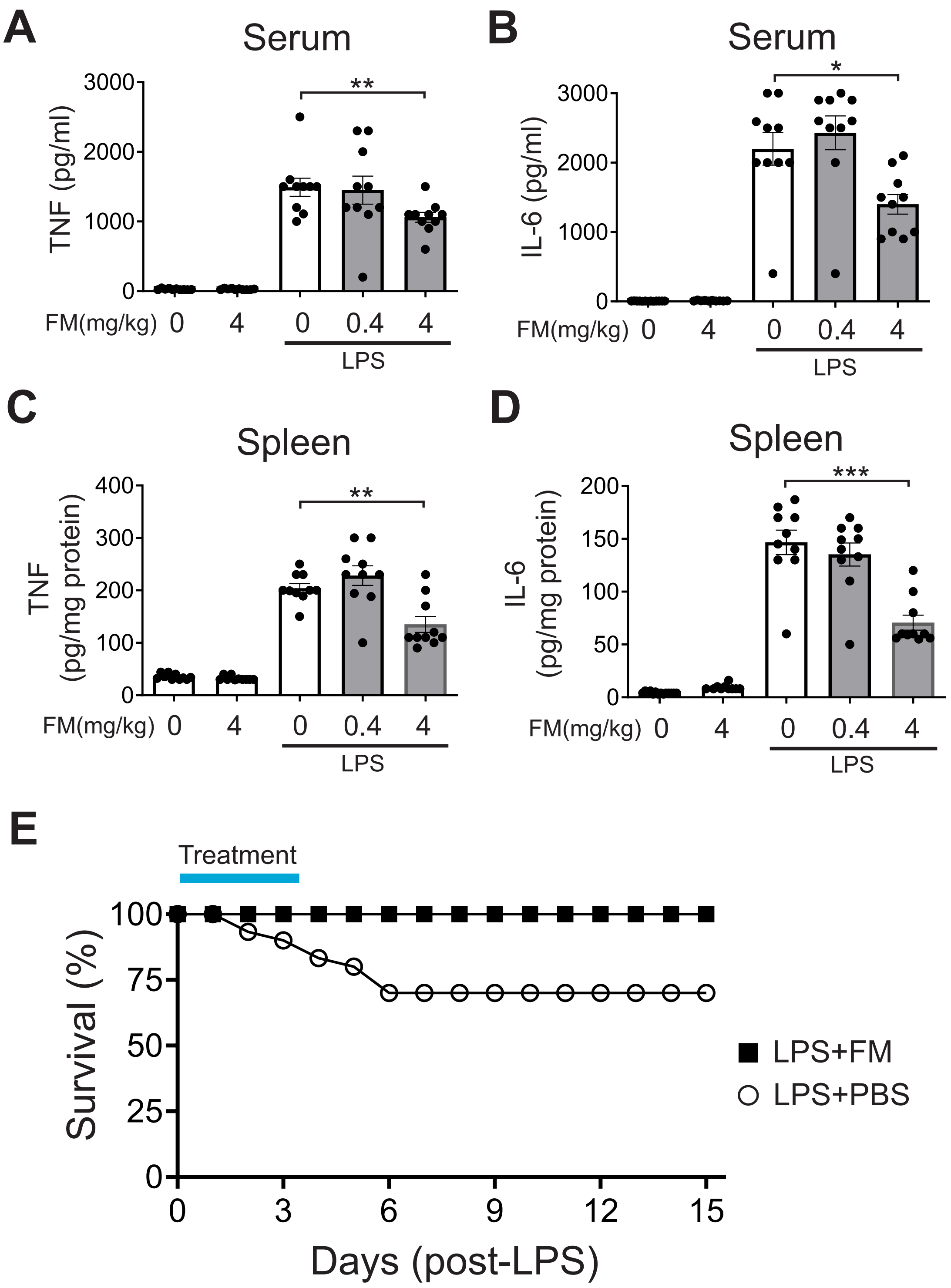
A
Serum
TNF (pg/ml)
3000
2000
1000
0
**
FM(mg/kg) 0 4 0 0.4 4
LPS
B
Serum
IL-6 (pg/ml)
3000
2000
1000
0
*
FM(mg/kg) 0 4 0 0.4 4
LPS
C
Spleen
TNF (pg/mg protein)
400
300
200
100
0
**
FM(mg/kg) 0 4 0 0.4 4
LPS
D
Spleen
IL-6 (pg/mg protein)
200
150
100
50
0
***
FM(mg/kg) 0 4 0 0.4 4
LPS
E
Treatment
Survival (%)
100
75
50
25
0
0 3 6 9 12 15
Days (post-LPS)
LPS+FM
LPS+PBS

Figure 2

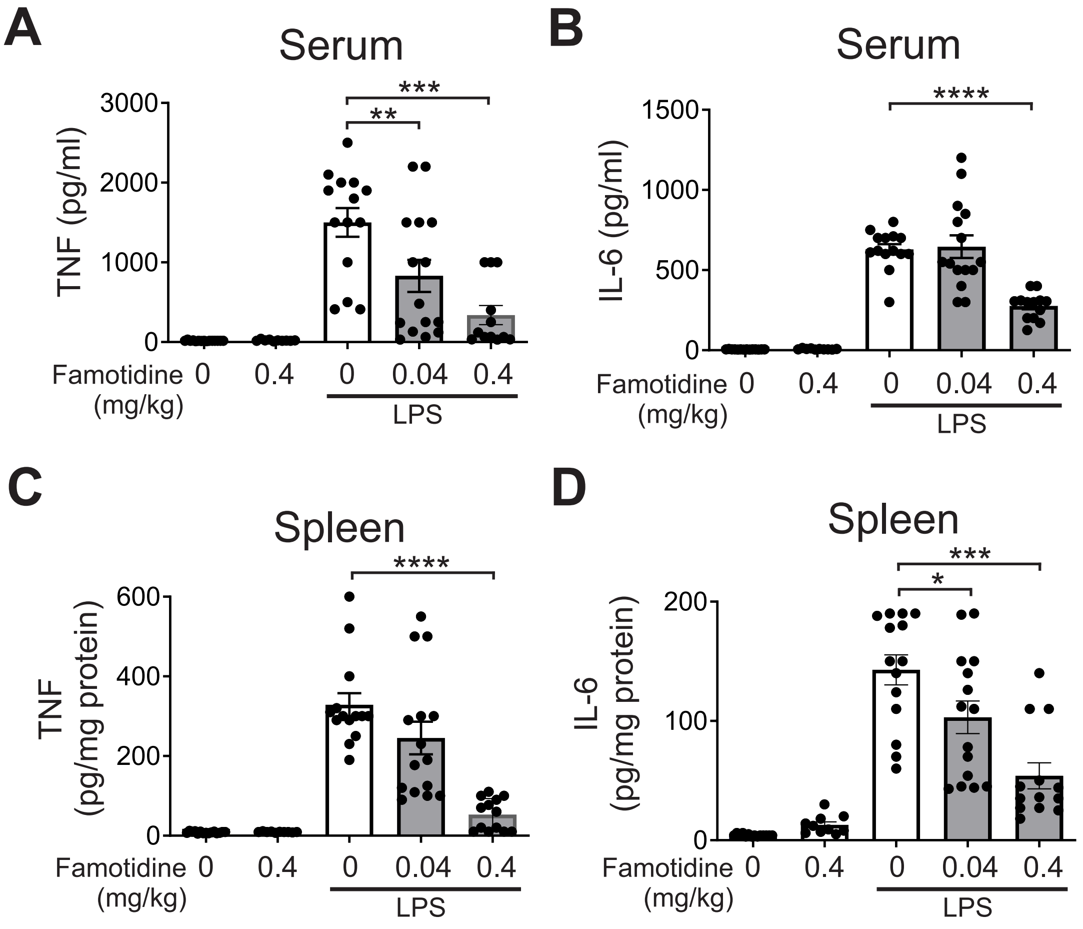

A
Serum
TNF (pg/ml)
3000
2000
1000
0
**
***
Famotidine (mg/kg)
0
0.4
0
0.04
0.4
LPS
B
Serum
IL-6 (pg/ml)
1500
1000
500
0
****
Famotidine (mg/kg)
0
0.4
0
0.04
0.4
LPS
C
Spleen
TNF (pg/mg protein)
600
400
200
0
****
Famotidine (mg/kg)
0
0.4
0
0.04
0.4
LPS
D
Spleen
IL-6 (pg/mg protein)
200
100
0
*
***
Famotidine (mg/kg)
0
0.4
0
0.04
0.4
LPS

Figure 3

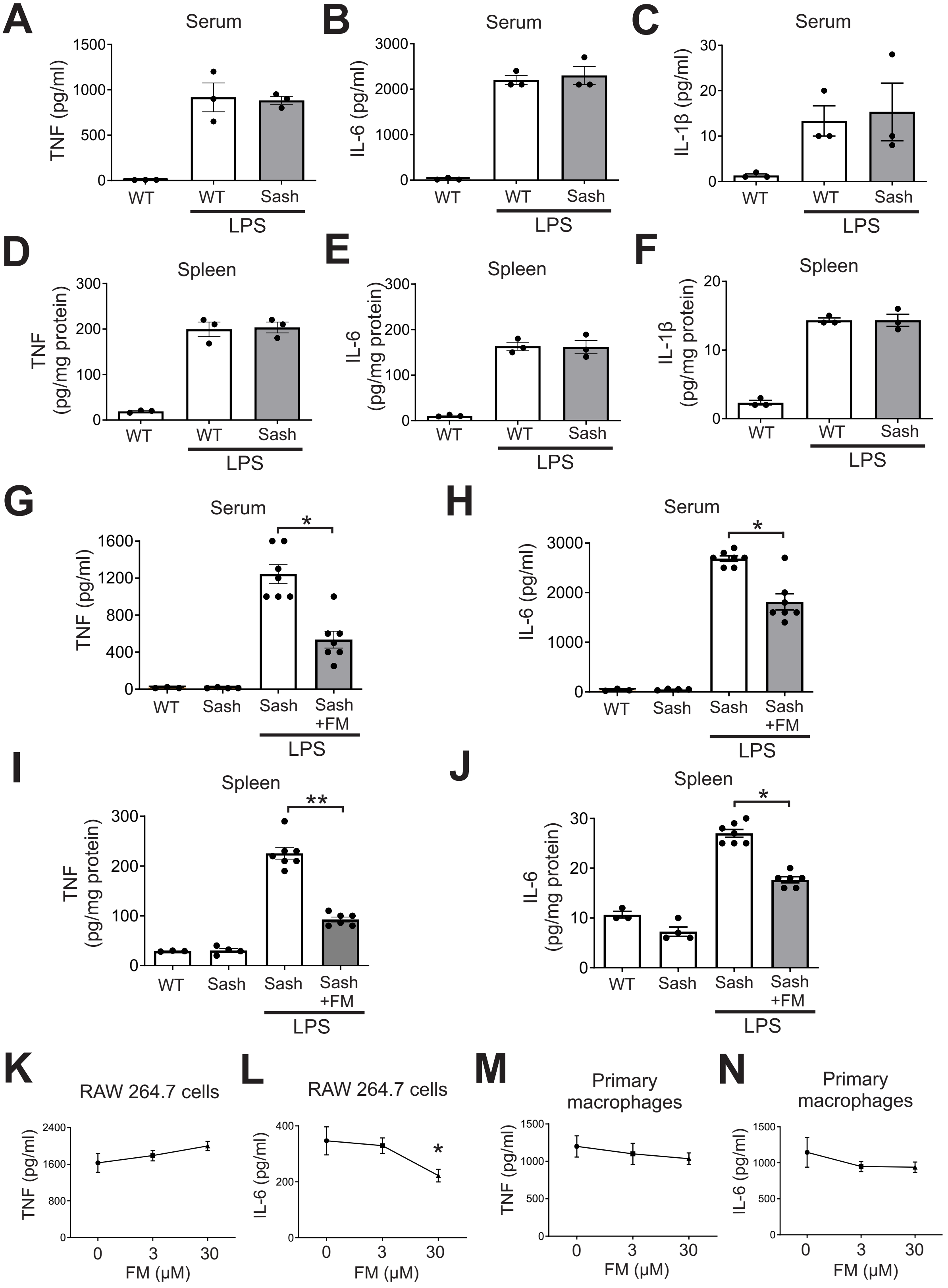
A
Serum
TNF (pg/ml)
WT
WT
Sash
LPS
B
Serum
IL-6 (pg/ml)
C
Serum
IL-1β (pg/ml)
D
Spleen
TNF (pg/mg protein)
E
Spleen
IL-6 (pg/mg protein)
F
Spleen
IL-1β (pg/mg protein)
G
Serum
TNF (pg/ml)
Sash +FM
H
Serum
IL-6 (pg/ml)
I
Spleen
TNF (pg/mg protein)
J
Spleen
IL-6 (pg/mg protein)
K
RAW 264.7 cells
TNF (pg/ml)
FM (μM)
L
RAW 264.7 cells
IL-6 (pg/ml)
FM (μM)
M
Primary macrophages
TNF (pg/ml)
FM (μM)
N
Primary macrophages
IL-6 (pg/ml)
FM (μM)

Figure 4

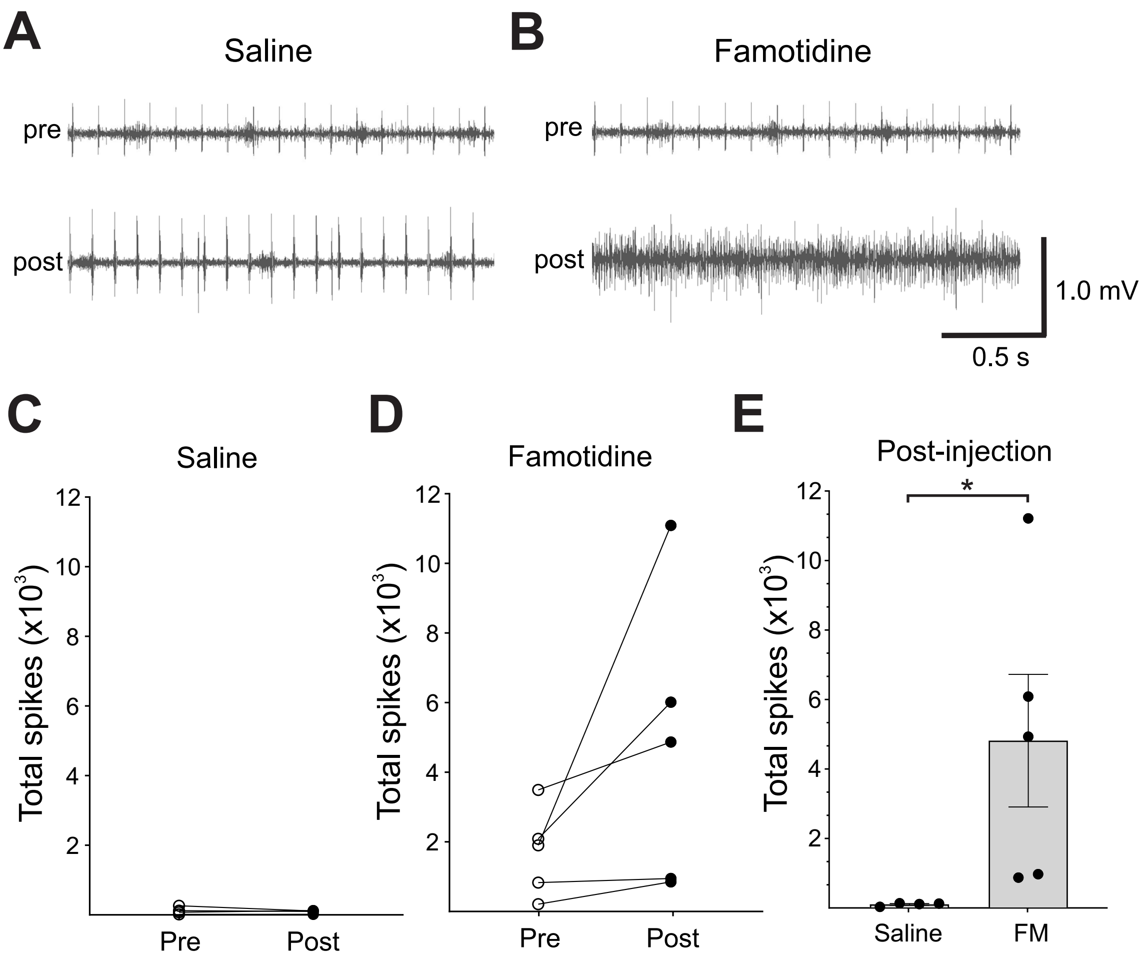
A
Saline
pre
post
B
Famotidine
pre
post
1.0 mV
0.5 s
C
Saline
Total spikes (x10³)
12
10
8
6
4
2
Pre
Post
D
Famotidine
Total spikes (x10³)
12
10
8
6
4
2
Pre
Post
E
Post-injection
*
Total spikes (x10³)
12
10
8
6
4
2
Saline
FM

Figure 5

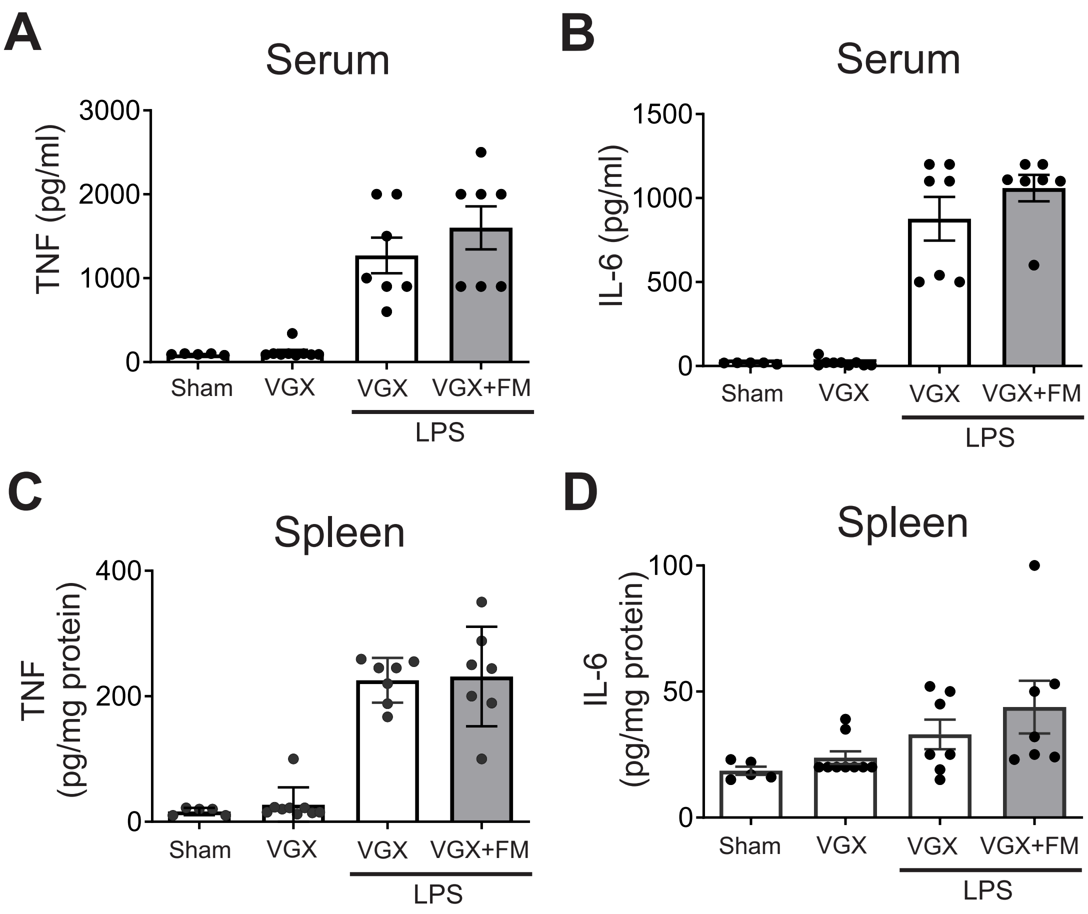
A
Serum
TNF (pg/ml)
3000
2000
1000
0
Sham
VGX
VGX
VGX+FM
LPS
B
Serum
IL-6 (pg/ml)
1500
1000
500
0
Sham
VGX
VGX
VGX+FM
LPS
C
Spleen
TNF
(pg/mg protein)
400
200
0
Sham
VGX
VGX
VGX+FM
LPS
D
Spleen
IL-6
(pg/mg protein)
100
50
0
Sham
VGX
VGX
VGX+FM
LPS

Figure 6

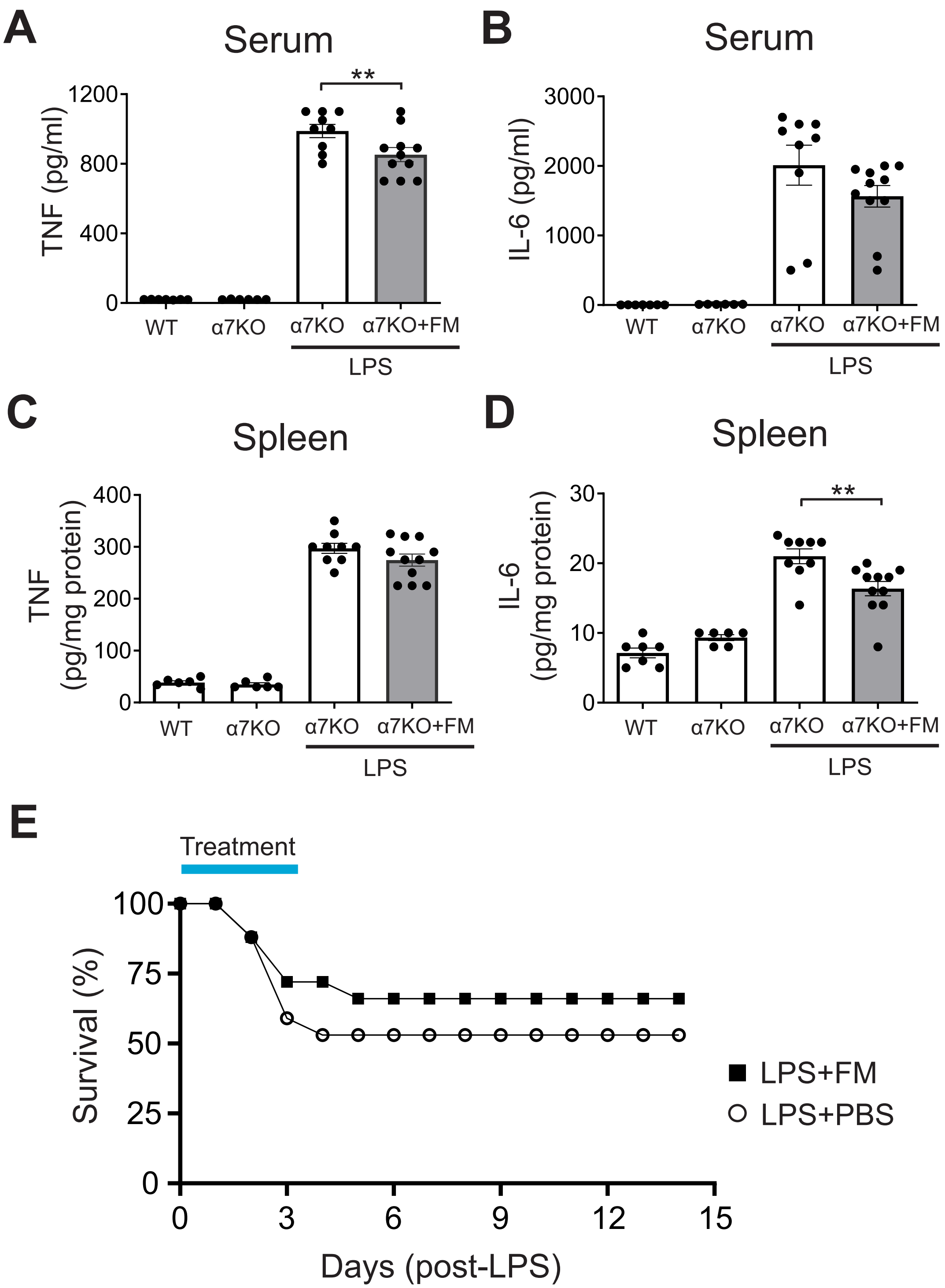
A
Serum
TNF (pg/ml)
1200
800
400
0
**
WT
α7KO
α7KO
α7KO+FM
LPS
B
Serum
IL-6 (pg/ml)
3000
2000
1000
0
WT
α7KO
α7KO
α7KO+FM
LPS
C
Spleen
TNF (pg/mg protein)
400
300
200
100
0
WT
α7KO
α7KO
α7KO+FM
LPS
D
Spleen
IL-6 (pg/mg protein)
30
20
10
0
**
WT
α7KO
α7KO
α7KO+FM
LPS
E
Treatment
Survival (%)
100
75
50
25
0
0
3
6
9
12
15
Days (post-LPS)
LPS+FM
LPS+PBS

Supplementary Figure 1

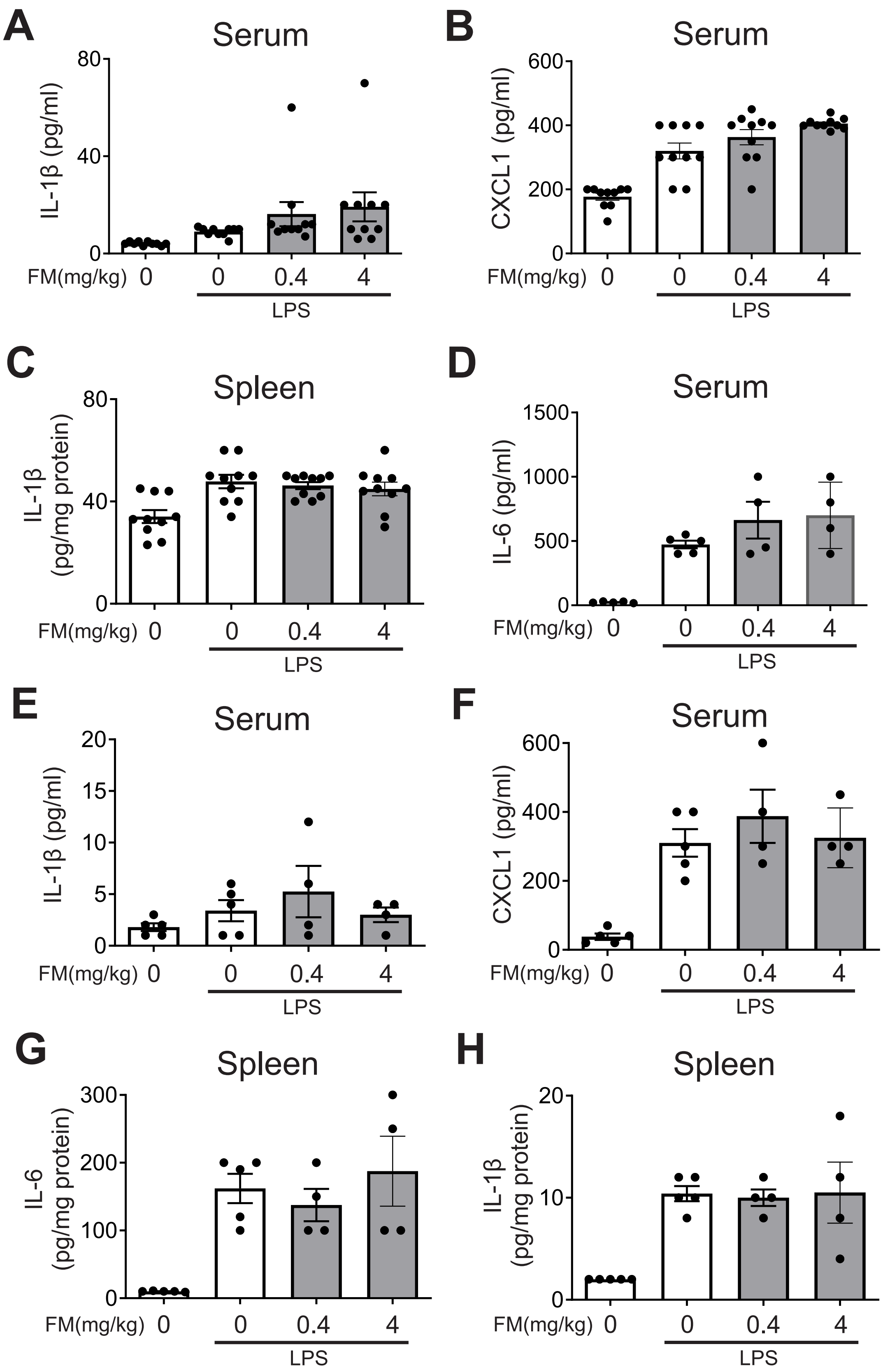

Supplementary Figure 2

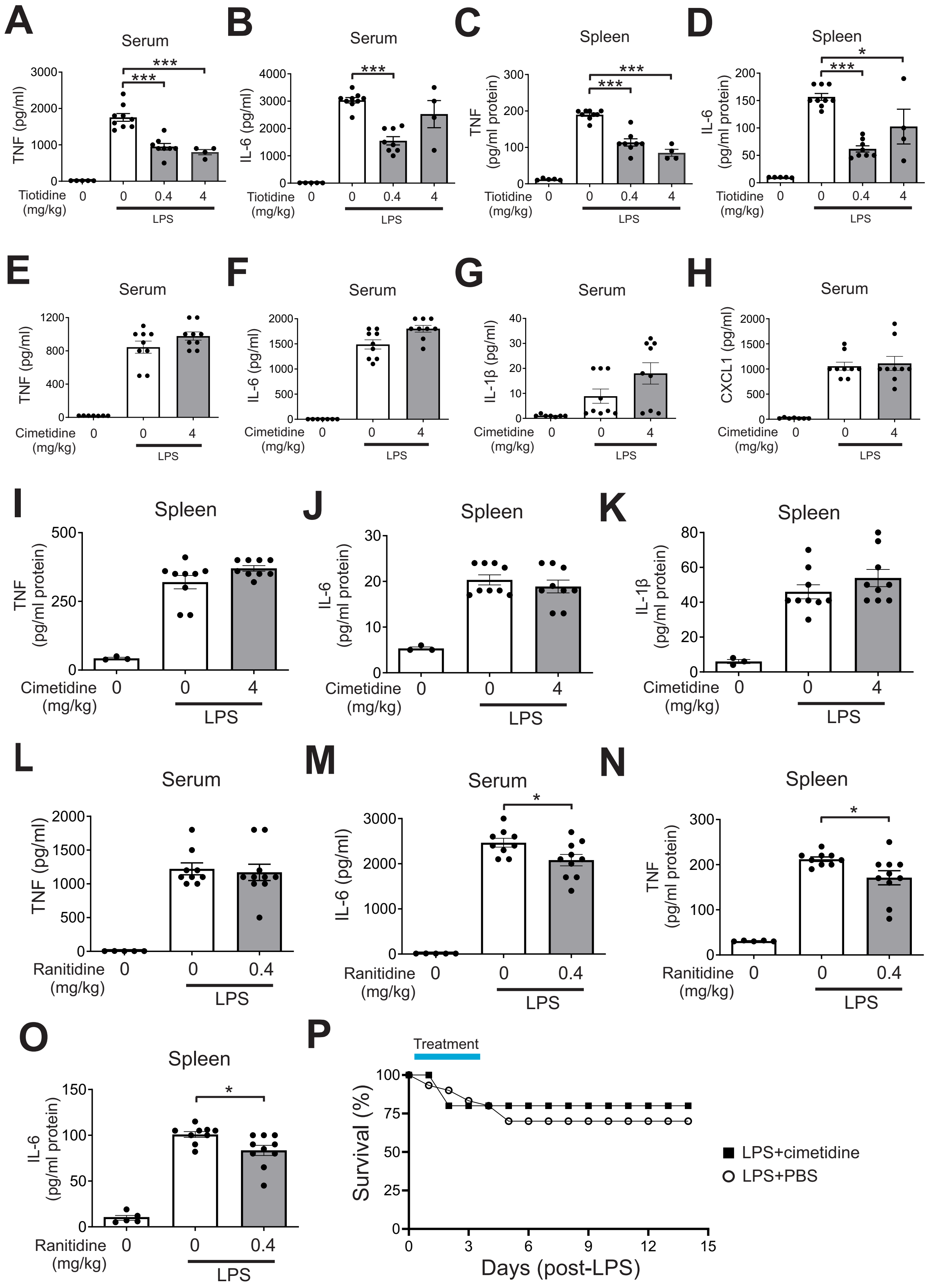

Supplementary Figure 3

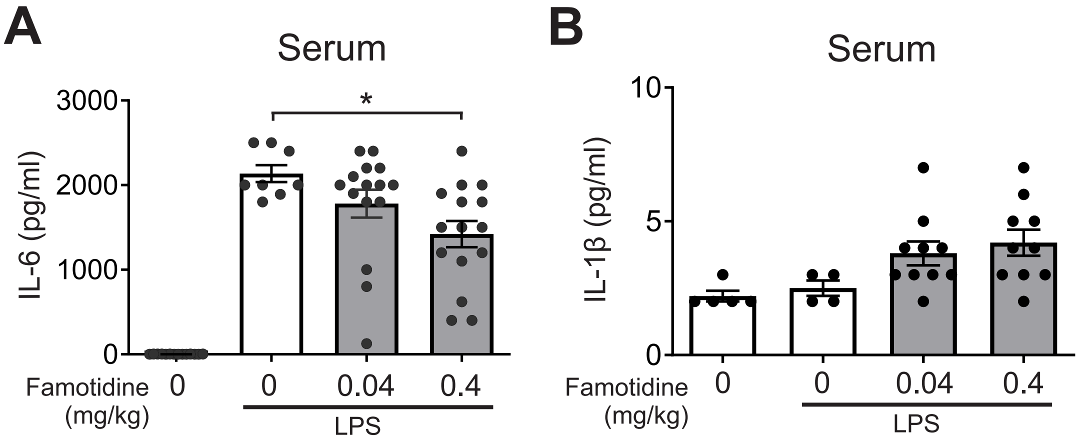

Supplement: Supplementary file 1 — Additional file 1: Figure S1. Intraperitoneal administration of famotidine did not significantly alter IL-1β or CXCL1 levels at 2.5 or 6 h post LPS exposure. A–D Male C57BL/6 mice, 8–12 weeks of age, were injected with LPS (7 mg/kg) with or without famotidine (FM, 0.4 or 4 mg/kg, in 100 µl volume), intraperitoneally 30 min before LPS injection. Mice were euthanized 2.5 h after LPS or FM administration. Serum and spleen IL-1β and CXCL1 were measured (N = 10 mice per group), as well as serum IL-6 (N = 5 mice per group). E–H Mice received an IP injection of famotidine or vehicle (0.04 or 0.4 mg/kg, in 5 µl volume) 30 min before LPS injection. LPS was administered IP at 7 mg/kg. Mice were euthanized 6 h post-LPS injection. N = 5 for normal group. N = 13–14 for others. Figure S2. Effects of other histamine 2 receptor antagonists. A–D Male C57BL/6 mice had ICV injection of PBS or tiotidine (0.04 or 0.4 mg/kg in 5 µl volume) 30 min before LPS. LPS was administered IP at 7 mg/kg. Mice were euthanized 2.5 h post-LPS injection and levels of serum and spleen TNF and IL-6 were measured. N = 5 for normal group. N = 4 for tiotidine 4 mg/kg group and N = 8 for other groups. *P < 0.02, ***P < 0.0001. E–K Male C57BL/6 mice, 8–12 weeks of age, received cimetidine or PBS (vehicle) via ICV injection 30 min prior to LPS (IP, 7 mg/kg). Mice were euthanized 2.5 h later and serum and spleen cytokines were measured. N = 3–6 for normal group, N = 9 for other groups. L–O Male C57BL/6 mice had ICV injection of PBS, ranitidine (0.4 mg/kg in 5 µl volume) 30 min before LPS. LPS was administered IP at 7 mg/kg. Mice were euthanized 2.5 h post-LPS injection and levels of serum and spleen TNF and IL-6 were measured. N = 5 for normal group, N = 8 for LPS + PBS and N = 10 for LPS + ranitidine group. *P ≤ 0.05. P Male C57BL/6 mice, 8–12 weeks old, were injected with LPS (6 mg/kg, IP). CM or PBS (4 mg/kg, in 100 µl volume) were injected intraperitoneally twice a day for 3 days, survival was monitored for [file 10020_2022_483_MOESM1_ESM.pdf]
